# GM-CSF regulates ILC states and myeloid cell signaling during ulceration in Crohn’s disease

**DOI:** 10.64898/2026.02.03.703526

**Published:** 2026-03-06

**Authors:** Joshua K. Morrison, Ksenija Sabic, Neha Maskey, Sayali Talware, Nai-yun Hsu, Colleen Chasteau, Elizabeth Aslinger, Jake T Herb, Shikha Nayar, Rachel Levantovsky, Christopher Tastad, Rachel Moss, Alan Soto, Monica Garcia-barros, Jessy Ntunzwenimana, Mariel Glass, Michelle Bao, Jiayu Zhang, Huajun Han, Jane Stevens, Lorena Tavares, Tin Htwe Thin, Sergey Khaitov, Alexander Greenstein, Rachel Brody, Jaime Chu, Arthur Mortha, Judy H Cho, Ling-shiang Chuang

## Abstract

Macrophage (M-), granulocyte (G-), and granulocyte–macrophage (GM-) colony-stimulating factors (CSFs) regulate myeloid cell function, yet their relative roles during inflammation remain poorly defined. To uncover how CSFs shape spatial immune niches in Crohn’s disease, we performed Xenium single-cell spatial transcriptomics on ileal tissues, revealing cell-type–specific expression and source–target interactions for each CSF. GM-CSF, unlike M-CSF or G-CSF, was locally enriched in ulcerated regions where lymphocytes adjacent to macrophage aggregates signaled through STAT5 phosphorylation. To study functional consequences, we developed a csf2rb ^−^ / ^−^ zebrafish model of intestinal injury. Using this model, we found that loss of GM-CSF signaling exacerbated epithelial damage and inflammation, whereas recombinant human GM-CSF limited injury by restraining ILC1 expansion, sustaining ILC3 maintenance, and promoting IL-22 production. Cross-species single-cell analysis revealed conserved ILC gene modules and GM-CSF–dependent transcriptional networks linking lymphoid and myeloid populations. These findings establish GM-CSF as a critical spatial regulator of myeloid–lymphoid crosstalk and intestinal immune homeostasis in Crohn’s disease.

## Full Text Availability

The license terms selected by the author(s) for this preprint version do not permit archiving in PMC. The full text is available from the preprint server.

